# SOCIAL DETERMINANTS, LONELINESS, DEPRESSION, AND CHRONIC ILLNESS FOR MIDLIFE AND OLDER ADULTS IN APPALACHIA

**DOI:** 10.1093/geroni/igae098.2879

**Published:** 2024-12-31

**Authors:** Laurie Theeke, Lashawn Hutto, LaTonya Smith

**Affiliations:** The George Washington University, Ashburn, Virginia, United States; George Washington University, Washington, District of Columbia, United States; George Washington University, Washington, District of Columbia, United States

## Abstract

Loneliness constitutes a significant health risk among midlife and older adults in Appalachia. This study presents a secondary data analysis exploring relationships among social determinants of health, loneliness, depression, and chronic illness. Common variable data from two prior studies that collected data from adults at a family health center was used for the analysis. The sample of 150 adults (88 women) ranged in age from 46 to 91years (mean 63.5, SD11.16) with mean loneliness score of 39.5 (SD11.13)- indicating high loneliness and averaged 3.2 (SD 1.88) chronic illnesses which increased with age. Over 40% were married with 74% living with others. An associate degree or higher was earned by 46%. Though 69.3% worked full-time, 43.3% reported income below $40,000 annually. Age inversely correlated with loneliness (p =.04) and depression scores (p <.001). Loneliness highly correlated with depression (p,.001) and inversely correlated (p <.01) with emotional, tangible, affective, positive, and total social support. Loneliness correlated with resting heart rate (p <.001) and body mass index (p=.05) but not with blood pressure. Men had higher loneliness (p=.027) and were more frequently diagnosed with hyperlipidemia, heart disease (p=.047), hypertension (p=.049), and stroke (p=.004). Women had higher scores for social support (p=.005) and functional ability. Findings highlight inequities in health for older adults, particularly men, living in Appalachia. Future studies are planned for larger cohort studies to better explicate psychophysiological pathways from loneliness to illness in this population and to examine the effectiveness of LISTEN, an intervention designed to target loneliness that demonstrated effectiveness in this population.

